# Nucleotide composition determines the role of translational efficiency in human genes

**DOI:** 10.6026/97320630013046

**Published:** 2017-02-28

**Authors:** Binata Halder, Arup Kumar Malakar, Supriyo Chakraborty

**Affiliations:** 1 Department of Biotechnology, Assam University, Silchar-788011, Assam, India;

**Keywords:** codon usage bias, tRNA adaptation index, gene expression

## Abstract

The basic sequence features were analysed that influence gene expression via codon usage bias of the selected forty coding sequences of Homo sapiens in a simple prokaryotic model i.e. E. coli K-12 genome. The prime objective was to elucidate the interrelationships among tRNA gene copy numbers, synonymous codons, amino acids and translational efficiency using tRNA adaptation index. It was evident from RSCU scores and principal component analysis, that only those preferred codons were used by the isoacceptor tRNAs that had G and C base at their third codon position. Relationship between tRNA adaptation index and amino acids, revealed that valine, arginine, serine and isoleucine showed significant positive correlation with gene expression. Therefore, it could be inferred that GC content in these genes might have the major role in shaping the codon bias and affecting the translational efficiency of the coding sequences.

## Background

Similar amino acid is encoded by multiple codons due to the degeneracy of the genetic code (excluding methionine and tryptophan), resulting in non-uniform usage of synonymous codons and this phenomenon is called codon usage bias (CUB). Codon usage bias is linked to innumerable features of gene expression and its efficiency and was observed in various prokaryotes and eukaryotes [1,2]. The magnitude to which any cellular protein is produced is generally determined by the stability of its translation, degradation, and dilution by cell growth. Research on codon usage with respect to the number of transfer RNA (tRNA) genes was done in various organisms and its incorporation into a simple model for quantitative protein production was discovered to improve the prediction of steadystate protein abundance [3]. Different codon–tRNA interactions are likely to vary among organisms per their effectiveness. The tRNA adaptation index (tAI) represents wobble interactions between codons and tRNA molecules and it is a measure of the adaptation of genes to the tRNA pool. It enables different tRNA species to spot a codon with different affinities [4]. Thus, tAI provides important information related to translation that is certainly not covered by other CUB measures. In the recent years, tAI was extensively used for studying problems in diverse biomedical disciplines like functional genomics, evolutionary biology, and systems biology [5]. Human protein-based therapeutics is the emerging area of drug development, primarily due to high sensitivity and specificity of these molecules that resulted in tremendous success rates in drug development. However, the integral complexity of proteins restricts their synthesis in living cells, by which production of recombinant proteins on a commercial scale becomes more expensive. Another drawback is that such proteins are not orally bioavailable as they get denatured or proteolyzed in the gut.

Thus, the cost of expensive heterologous human protein production may be reduced by incorporating methods of codon usage bias and codon optimization to make synthetic genes. Because of its fast growth rate and other famous genetic uniqueness E. coli has been widely used to produce recombinant proteins [6]. The newly synthesized human protein in E. coli cells come across several translational errors such as amino acid substitution, stalling, termination, and possibly frame-shifting. And this occurs when the codon bias of the human genes and E. coli differ from each other [6]. Therefore, we hypothesize to obtain a significant correlation between tRNA levels, and other sequence features related to the translational activity, wherein the coding sequences are analyzed to improve the expression efficiency. These sequence features include gene expression level [7], gene length [8], protein amino acid composition [9], tRNA abundance [10], mutation frequency and patterns [11], and GC composition [12], tRNA adaptation index (tAI) and amino acid frequencies.

This study is purported to predict the expression levels of forty human genes that are of medical importance in E. coli K-12 genome. It also focuses on the relationship between the codon usage of human genes and the tRNA genes (as tRNA molecules play a vital role in transporting the anti-codons to pair with its respective codons) of E. coli K-12. We used tAI as a prime measure to investigate the role of tRNA in protein expression and to estimate the gene expression levels.

## Methodology

### Data retrieval

Forty coding sequences (CDS) of human genes were retrieved from NCBI (http://www.ncbi.nlm.nih.gov) database to analyze the relevant factors of nucleotide contents and synonymous codon usage patterns, provided in the supplementary sheet.

### Relative Synonymous Codon Usage (RSCU)

It is a simple measure of the heterogeneity in the usage pattern of synonymous codons [13]. RSCU value greater than one indicates that the codon is over-represented and vice versa [14].

$\mathrm{RSCUij}=\frac{\mathrm{Xij}}{\frac{1}{\mathrm{ni}}\overset{\mathrm{ni}}{\underset{\mathrm{j=1}}{\Sigma }}\mathrm{Xij}}$

Where Xij is the frequency of the jth codon for the ith amino acid and ni being the number of alternative synonymous codons available for the ith amino acid.

### tRNA adaptation index

It is measured relative to the supply of the tRNAs that are required for translation of codons to amino acids. The tRNA availability is the driving force for translational selection. The tRNA adaptation index estimates the extent of adaptation of a gene (cds) to its genomic tRNA pool. It is a measure used for predicting gene expression [4].

${\mathrm{tAI}}_{g}={\left(\overset{l_{g}}{\underset{\mathrm{k=1}}{\prod }}\mathrm{Wik}\right)}^{\frac{1}{l_{g}}}$

Where lg is the length of the gene in codons, and Wik is the relative adaptiveness value of the codon.

### Aromaticity and Isoelectric point (pI) of proteins

Aromaticity is the relative frequency of aromatic amino acids in a protein [15] in the translated gene product. Isoelectric point of an amino acid is the pH at which the amino acid doesn’t migrate in an electric field. The values of pI represent the zwitterionic effect on the amino acids.

### Principal component analysis (PCA) and Correspondence analysis (COA)

PCA is a multivariate statistical method for simplifying the multidimensional information of the data matrix into a twodimensional map [16]. Usually the first and the second principal components contain maximum information.

COA identifies the major trends in the variation of the data and distributes the genes along continuous axes, with each subsequent axis explaining a decreasing amount of the variation [17].

### Software used

All the codon usage bias and the base compositional analyses were performed using an in-house PERL program developed by SC (corresponding author). PCA was done by XLSTAT. COA of amino acid usage was performed using CodonW software. Heatmap based on the relative frequencies of codons and amino acids were plotted using software package XLSTAT. Codon usage variation was analyzed based on RSCU value for each synonymous codon using XLSTAT. SPSS - version 21.0 was used to make other graphical representations.

### Statistical analysis

Microsoft Excel was used to perform the basic data analysis and interpretation. Pearson’s correlation and statistical test of significance were performed (p<0.01 and p<0.05) using SPSS - version 21.0.

## Result and discussion

The prime goal of the study was to analyze the codon usage patterns, to predict the expression level of the proteins (Table 1), and to evaluate the degree of heterogeneity in codon usage.

### Base compositional dynamics

The human coding sequences were analyzed to calculate individual nucleotides, as well as GC and AT content at the third position of the synonymous codons. The observed pattern of base compositions is presented in the supplementary sheet. These human genes were abundant in guanine (27.34%) and cytosine (25.77%) followed by adenine (24.67%) and thymine (22.22%). Similarly, the average percentage of GC (53.12%) was found to be higher than that of AT (46.87%). It was reported that high GC content is a consequence of a GC-biased repair of mismatches during recombination [18]. GC contents at three codon positions (GC1, GC2, and GC3) were calculated The GC3 and GC1 contents were 62.83% and 55.85%, respectively. In human, the GC content of large genome fragments (isochores) ranges from 30% to 60% and the GC content at the third codon position (GC3) ranges from 25% ≥ 90% [19].

### GC3 on codon bias

**Figure 1:**
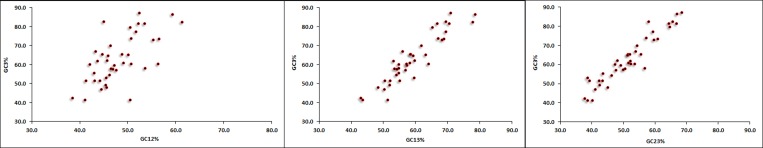
GC content against GC12, GC23 and GC13 for the coding sequences of Homo sapiens

To examine the association between codon usage bias and mutational pressure in these genes, we correlated GC3 with GC12, GC23, and GC13, where GC12 is the average of GC1 and GC2, similarly, GC23 is the average of GC2 and GC3, GC13 is the average of GC1 and GC3. From the Fig. 1, it was observed that GC3 showed significant positive correlation with GC23 (r=0.948, p<0.01), GC13 (r=0.927, p<0.01) and GC12 (r=0.651, p<0.01), which indicated that similar effect of GC mutation bias on the three positions of codons. Codon usage might have been subjective to an underlying bias in the dinucleotide usage [20]. Hence it can be inferred that codon bias in the selected human genes, is more likely to be characterized by mutation bias. However, to predict the expression level of the genes, %GC at three codon positions was correlated with tAI. We observed weak correlation of tAI with GC1 (r= -0.07), GC2 (r= -0.10) and GC3 (r= -0.11), respectively. This indicated that GC content is not a good predictor of gene expression. In conformity with our study, GC3 content was also found to be a very poor predictor of human gene expression in E. coli K-12 [21]. This reveals that high %GC content may not be required for the efficient expression of genes in E. coli and indicates that besides %GC there might be other forces affecting human gene expression in E. coli genome. Bernardi, 1993 observed that codon usage bias is mainly due to the difference in the patterns of GC content found in the human genome [22]. Association between codon usage bias and GC content in the surrounding non-coding region could be taken as a support for directional mutational pressure [23].

### Codon usage pattern

**Figure 2:**
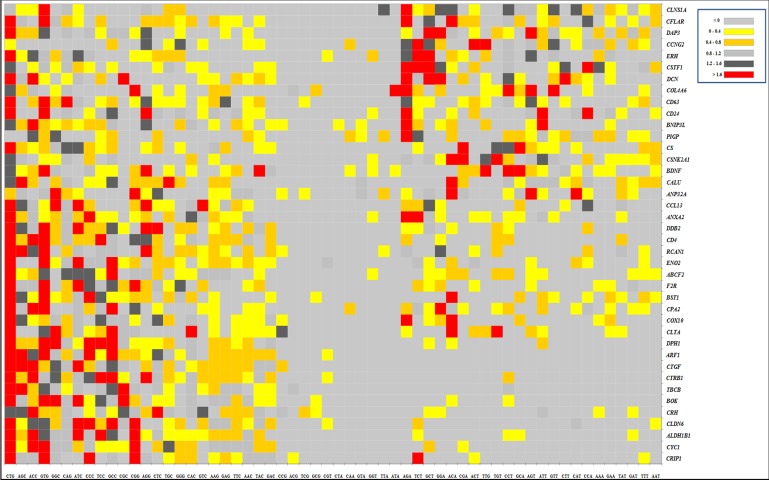
RSCU values of the synonymous codons encoding each amino acid have been plotted against each coding sequence

The codon usage pattern of the forty human coding sequences was investigated by calculating RSCU values (Table 2). It was found that the preferred codons (RSCU > 1.6) are biased strongly towards G/C bases in the third position supporting the findings of Dass and Sudandiradoss 2012 [24]. The over-represented codons (RSCU>1.6) have been depicted in red color, and the under-represented ones (RSCU<1.6) are in black to light grey colours (Figure 2). Most of the preferred codons end in G or C, thus GC3 content might play a key role in gene expression, as also studied by Duret and Mouchiroud 1999 [25].

### Principal Component Analysis (PCA)

**Figure 3:**
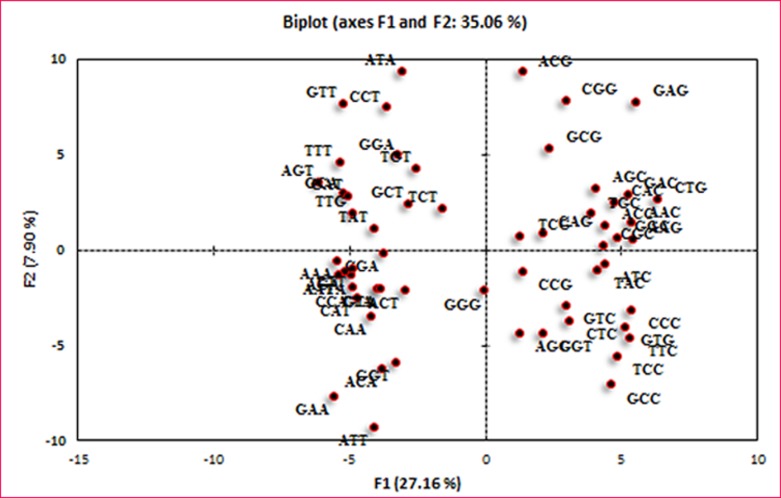
PCA analysis shows the variation amongst the RSCU values of codons in the genes

To further test if mutation bias is the consequence of codon usage bias, we performed PCA using RSCU values of all codons. The GC and AT bases should be uniformly used due to mutation bias in coding sequences. In paradox to this statement, natural selection for codon choice should not cause the proportional use of GC and AT, since tRNA isoacceptors show an important role in modeling the codon usage bias, thereby influencing gene expressivity [26]. From this analysis, it was evident that the coding sequences did not show uniform usage of GC and AT base pairs which further indicated that natural selection might have played a key role in determining gene expression (Supplementary sheet). The pattern of codon usage in human genes was revealed in Figure 2. PCA revealed that the first axis (F1) accounted for 27.16% and a second axis (F2) accounted for 7.90% of the total variation in the codon usage. Furthermore, the synonymous codons having positive F1 values showed maximum usage of G or C at the synonymous third codon position (except GGG, TTG). But negative F1 values of codons revealed that the codons were enriched with A or T in the third position (except CGT) Figure 3.

### Amino acid usage and gene expression

The correlation analysis between tAI and different amino acids revealed that the amino acids Val (r=0.67, p<0.01), Arg (r=0.65, p<0.01), Ser (r=0.65, p<0.01) and Ile (r=0.64, p<0.01) showed highly significant positive correlation with tAI. This suggests that the amino acids could influence tAI i.e. gene expression through the corresponding optimal codon-anticodon pairing.

The codon-anticodon combination in these genes seemed to be constrained by tRNA availability due to limited copy numbers in E. coli genome as compared to the human genome. We observed that preferred codons correspond to the most abundant tRNA species and similar results were also reported by Ikemura [27]. In another study, Zhang et al. (1991) [28], showed that in E. coli, S. cerevisiae, D. melanogaster, and primates (mainly Homo sapiens) the proteins with a high percentage of low-usage codons can be considered as cases, wherein, excess of the protein may possibly be detrimental. In bacteria Saier (1995) showed evidence that the translation of proteins involved in some specific functions may be regulated by using rare codons [29]. Our results indicate that the translation efficiency of human genes in a heterologous system like E. coli K-12 genome may function at the expense of the demand for tRNA molecules carrying specific amino-acids and the supply of matching codons by the coding sequences on ribosomes [30].

Correspondence analysis (COA) of amino acid usages revealed a single major axis of variation in the genes. Axis 1 revealed an insignificant correlation with GRAVY, aromaticity, hydrophilicity, molecular weight of proteins, gene expressivity measure (CAI), the effective number of codons and tAI. However, the isoelectric point of the proteins indicated moderately negative correlation with the axis 1 (Table 2). The correlation analysis of axis 1 with pI suggests that amino acid usage may poorly affect the surface charge distribution of proteins with the prevalence of acidic and basic residues in these sequences.

## Conclusion

This study was undertaken to comprehend the role of base composition on translational efficiency of human genes in E. coli and it revealed that GC3 content is a poor predictor of gene expression (tAI). The present study reveals that although the protein composition is determined by GC richness in the genes, other factors like RSCU, tAI and pI of the proteins. However, the codon usage variation was constrained by compositional pattern. Principal component analysis also supported that most codons showed biased effect on guanine and cytosine at the third codon position and the preferred codons usually end with either G or C. Positive significant correlation of gene expression parameter with a few amino acids viz. Val, Arg, Ser, Ile indicated that amino acids composition might influence the gene expression. Only pI showed moderate negative correlation with axis 1 of the correspondence analyses. It could be stated that mutation might have played a major role in predicting the level of gene expression. This study has provided a basic understanding of the causes for codon usage bias, which could be beneficial for further studies in molecular evolution, cloning and heterologous human protein production for therapeutic usage.

## Conflict of Interest

There is no conflict of interest in this research.

## Figures and Tables

**Table 1:** List and functions of the forty human genes with their accession numbers (http://www.genecards.org/) included in the study

Cds No.	Accession Number	Gene symbol	Functions
1	AF400438.1	BDNF	Differentiation of a few neuronal populations of the peripheral and central nervous systems
2	BT007436.1	ANP32A	Proliferation, differentiation, caspase-dependent and independent apoptosis, suppression of tumour, regulation of mRNA trafficking
3	BT007393.1	ARF1	Modulates vesicle budding and un-coating within the Golgi complex
4	BT007418.1	ALDH1B1	Metabolism of corticosteroids, biogenic amines, neurotransmitters, and lipid peroxidation
5	BT007432.1	ANXA2	Calcium and phospholipid binding protein, calpactin 1, involved in exocytosis, overexpressed in small cell lung carcinoma
6	BT007451.1	ABCF2	ATP-binding cassette superfamily, subfamily F (GCN20)
7	BT019501.1	BNIP3L	Pro-apoptotic, inhibiting growth of cancer cells, widely expressed in target of the mitochondria
8	BT007272.1	BOK	Induces apoptosis in a manner dependent on BAX and BAK
9	BT019502.1	BST1	Involved in pre-B-cell growth, expressed in various tissues
10	BT020143.1	CALU	Involved in regulation of vitamin K-dependent carboxylation of multiple N-terminal glutamate residues
11	BT007403.1	CPA2	Similar to that of carboxypeptidase A, but with a preference for bulkier C-terminal residues
12	BT019792.1	CSNK2A1	Regulation of cell growth RaF/MAPK signalling, as a free CK2 beta
13	BT006751.1	CFLAR	Crucial link between cell survival and cell death pathways in mammalian cells. Inhibitor of TNFRSF6 mediated apoptosis
14	BT019811.1	CD4	Receptor for human immunodeficiency virus-1
15	BT007404.1	CD24	Promotes AG-dependent proliferation of B-cells, and prevents their terminal differentiation into antibody-forming cells
16	BT020138.1	CD63	Promotes cell survival, reorganization of the actin cytoskeleton, cell adhesion, spreading and migration, via its role in the activation of AKT and FAK/PTK2
17	BT007385.1	CCL13	Accumulation of leukocytes at both sides of allergic and non-allergic inflammation
18	BT019907.1	CLNS1A	Splicing of cellular pre-mRNAs
19	BT007356.1	CTRB1	Cleavage at: Tyr- -Xaa, Trp- -Xaa, Phe- -Xaa, Leu- -Xaa
20	BT007414.1	CS	Catalysing the first step of citric acid cycle
21	BT007170.1	CLTA	Coated vesicles and coated pits, translocation to plasma membrane through AP2 binding
22	BT007399.1	CLDN6	Tight junction-specific obliteration of the intercellular space
23	BT007138.1	CSTF1	Polyadenylation and 3-end cleavage of mammalian pre-mRNAs
24	BT007279.1	F2R	Stimulate phosphoinositide hydrolysis and may play a role in platelets activation and in vascular development
25	BT007228.1	COL4A6	Structural component of glomerular basement membranes
26	BT019794.1	CTGF	Promotes proliferation and differentiation of chondrocytes
27	BT007453.1	CRH	Regulates the release of corticotropin from pituitary gland
28	BT006985.1	COX10	Converts protoheme IX and farnesyl diphosphate to heme O
29	BT019503.1	CCNG2	Growth regulation and in negative regulation of cell cycle progression
30	BT007181.1	CRIP1	Intracellular zinc transport protein, cellular repair
31	BT019798.1	CYC1	Electron transporter, transferring electrons from CoQH2-cytochrome c reductase complex and cytochrome c oxidase complex activity
32	BT007424.1	TBCB	Involved in regulation of tubulin heterodimer dissociation
33	BT007139.1	DDB2	Damage specific DNA binding protein involved in nucleotide excision repair
34	BT019494.1	DAP3	Mediates interferon gamma induced apoptosis, TNF and TNFRSF6 induced cell death
35	BT019800.1	DCN	Involved in the differentiation of retinal glanglion cells
36	BT019878.1	DPH1	Post-translational modification of histidine. Acts as a tumour suppressor in lung and breast cancers
37	BT007363.1	RCAN1	Inhibits calcineurin-dependent transcriptional responses by binding to the catalytic domain of calcineurin A.
38	BT007053.1	PIGP	Catalysing the transfer of N-acetylglucosamine from UDP-N-acetylglucosamine to phosphatidylinositol,the first step of GPI biosynthesis
39	BT006877.1	ERH	Methyl-CpG binding, poly(A) RNA binding
40	BT007383.1	ENO2	Nephic and neuroprotective property on a broad spectrum of central nervous system (CNS) neurons.

**Table 2:** Amino acid properties, tAI, CAI with the major axis (Axis 1) of correspondence analysis

Axis 1 (COA)	Gravy	Hydrophilicity	Aromaticity	pI	MW (dalton)	tAI	CAI (Sharp)
0.00412	-0.46	0.15	0.07	9.01	27273.52	1.17	0.7945
0.09822	-1.26	0.94	0.04	3.99	28313.17	1.1	0.8551
0.67894	-0.26	-0.01	0.09	6.31	20288.42	1.1	0.5128
0.15079	-0.24	0.01	0.09	6.36	56013.3	1.15	0.872
0.03745	-0.52	0.28	0.08	7.58	38059.6	1.15	0.825
0.19049	-0.5	0.2	0.06	6.95	67887.17	1.15	0.9381
0.13343	-0.73	0.2	0.05	5.52	22433.02	1.13	0.792
0.00005	0.31	-0.14	0.08	9.33	22599.64	1.11	0.5017
-0.1463	-0.22	-0.02	0.09	7.97	34771.17	1.18	0.8292
-0.07186	-1	0.54	0.11	4.47	35745.53	1.15	0.8246
0.43945	-0.22	-0.08	0.11	5.77	45122.56	1.17	0.8863
0.03454	-0.48	0.04	0.1	7.29	42965.56	1.17	0.8846
0.10988	-0.35	0.14	0.08	8.19	53302.24	1.16	0.8959
0.09954	-0.26	0.01	0.06	9.6	50157.74	1.15	0.8929
-0.08587	0.35	-0.47	0.04	9.69	7947.1	0.99	0.246
-0.34632	0.77	-0.43	0.09	8.14	25364.49	1.13	0.7534
-0.13704	-0.05	-0.1	0.07	9.94	10713.94	1.04	0.2209
-0.23608	-0.66	0.32	0.08	3.97	25398.59	1.13	0.7926
0.24812	0.16	-0.24	0.08	6.79	27461.68	1.14	0.5346
-0.2211	-0.18	-0.19	0.1	7.8	28095.49	1.18	0.769
-0.00083	-0.81	0.41	0.06	4.45	23389.67	1.14	0.8002
0.15709	0.69	-0.54	0.08	8.33	23005.37	1.13	0.7225
-0.02687	-0.33	-0.01	0.09	6.12	46588.09	1.19	0.9303
-0.21032	0.47	-0.45	0.13	8.62	46924.2	1.16	0.8927
-0.13422	0.06	-0.22	0.08	6.03	7786.17	0.92	0.3915
-0.2663	-0.21	0.1	0.06	8.36	37661.31	1.14	0.7817
-0.18366	-0.33	0.06	0.04	10	20877.28	1.12	0.5366
-0.46035	0.13	-0.29	0.1	9.3	47657	1.17	0.9419
-0.00638	-0.08	0.05	0.07	5.33	37504.68	1.16	0.8366
-0.17796	-0.81	0.21	0.1	9.05	7852.22	1.02	0.1405
-0.1186	-0.14	-0.06	0.08	9.15	34060.78	1.16	0.8213
0.12759	-0.54	0.27	0.1	5.06	27053.32	1.14	0.7069
-0.13524	-0.38	-0.03	0.08	9.56	45822.12	1.17	0.9006
0.02823	-0.44	0.02	0.09	9.02	43252.29	1.17	0.8911
0.01326	-0.25	-0.02	0.06	8.75	38521.67	1.17	0.9175
-0.11827	0	-0.1	0.08	6.01	38815.01	1.16	0.8419
0.06471	-0.52	0.16	0.1	5.99	21683.94	1.12	0.6554
-0.02952	0.29	-0.53	0.16	5.24	15271.86	1.11	0.5271
0.11713	-0.67	0.12	0.11	5.63	11986.72	1.01	0.4237
0.12555	-0.19	0.1	0.07	4.91	46451.88	1.15	0.8931
Correlation between Axis 1	-0.178	0.185	-0.024	-0.340*	0.099	0.032	0.019
				p<0.05
